# Quality of Life for Obese Women and Men in Turkey

**Published:** 2007-06-15

**Authors:** Fulden Saraç, Sebnem Parýldar, Erdal Duman, Fusun Saygýlý, Mehmet Tüzün, Candeger Yýlmaz

**Affiliations:** Ege University Hospital; Department of Psychiatry, Ege University, Izmir, Turkey; Department of Endocrinology and Metabolism, Ege University Hospital, Izmir, Turkey; Department of Endocrinology and Metabolism, Ege University Hospital, Izmir, Turkey; Department of Endocrinology and Metabolism, Ege University Hospital, Izmir, Turkey; Department of Endocrinology and Metabolism, Ege University Hospital, Izmir, Turkey

## Abstract

**Introduction:**

Obesity is a complex, multifaceted disease that is widespread and growing in the developing world. People who are obese experience health-related quality-of-life impairments.

**Methods:**

We administered the SF-36 Health Survey questionnaire to 1752 obese adults and 400 normal-weight adults in Izmir City, Turkey. We then compared the mean scores of the two groups by sex in eight quality-of-life domains.

**Results:**

Differences in scores between obese women and normal-weight women were statistically significant in seven of eight SF-36 domains; differences in scores between obese men and normal-weight men were statistically significant in six of eight domains. Obese women were significantly more impaired than obese men in four of eight domains. Among obese women, 45.0% experienced a reduced quality of life, compared with only 13.2% of normal-weight women. Similarly, 41.3% of obese men experienced a reduced quality of life, compared with only 9.3% of normal-weight men.

**Conclusion:**

Obesity is associated with poor levels of health, particularly poor levels of physical and social well-being.

## Introduction

Obesity is a complex, multifaceted disease that is widespread and growing in the developing world. People who are obese experience health-related quality-of-life impairments. Impairment in an obese individual's capacity to live as fully and actively as he or she desires may be as serious a consequence of obesity as its adverse effects on morbidity and mortality (1). Both physical and psychosocial functioning have been shown to be negatively affected by excess weight; greater impairments have been associated with greater degrees of obesity (2).

In 1947, the World Health Organization defined *health* as both the absence of disease and infirmity and the presence of physical, mental, and social well-being (3). Health-related quality of life refers to well-being in the physical, psychological, and social domains; well-being in each domain can be assessed by measuring an individual's objective functioning and subjective perceptions of health. Quality-of-life assessments can be used to measure and compare the effectiveness of different treatments and to evaluate the impact of a treatment on how patients feel and function in their everyday lives (4).

Until recently, there has been little standardization of quality-of-life measures among people who are obese; many researchers have simply developed their own set of nonvalidated questions. The field of quality-of-life research has grown, however, and standards for developing and validating quality-of-life instruments have been proposed (4-11). The SF-36 Health Survey (QualityMetric Inc, Lincoln, RI) has been used to study obesity because it is comprehensive, brief, psychometrically sound, and consistent with current guidelines for health-related quality-of-life instruments (4,11-15).

Our aim in this study was to compare the health-related quality of life of obese and normal-weight adults in Turkey. In addition, we sought to describe the conditions contributing to poor health-related quality of life among obese patients. We hypothesized that obesity negatively affects both physical and psychosocial functioning.

## Methods

### Study population 

The study population consisted of two groups of adults aged 20 to 65: an obese group of 1752 (254 men and 1498 women) and a normal-weight group of 400 (150 men and 250 women). The obese participants were all patients at an obesity clinic operated by the Department of Endocrinology and Metabolism at Ege University in Izmir City, Turkey; patients with associated comorbidities such as hypertension, diabetes mellitus, or cardiovascular disease were excluded from the study. The group of normal-weight men and women were recruited from the general outpatient population of the Department of Endocrinology and Metabolism at Ege University.

Diagnosis of obesity was determined by measurement of body mass index (BMI [weight in kg/height in m^2^]). Those in the obese group had a BMI greater than or equal to 30.0; those in the normal-weight group had a BMI less than 25.0. All participants signed an informed consent form, and the Ege University Hospital ethics committee approved the study.

### Study design 

To assess participants' health-related quality of life, we used the Medical Outcomes Study Short Form-36 Health Survey (SF-36) developed by Ware et al (12). We translated the questionnaire into the Turkish language. Responses were included in the analysis only if all domains of the questionnaire were answered in full. The questionnaire took an average of 20 minutes to complete (range, 15–45 min). The survey was conducted from November 2004 through December 2005.

### The SF-36 questionnaire 

The SF-36 questionnaire is a self-evaluation instrument consisting of 36 items, including 35 items in eight domains that provide a scaled assessment of respondents' quality of life during the previous 4 weeks. Ten items address the domain of physical functioning, defined as limitations in physical activities such as bathing or dressing because of health problems; four items address the domain of *role-physical* limitations, defined as limitations in usual activities such as work or other daily activities because of physical health problems; two items address the domain of bodily pain; five items address the domain of general health; four items address the domain of vitality (energy and fatigue); two items address the domain of social functioning, defined as limitations in social activities because of physical or emotional problems; three items address the domain of *role-emotional *limitations, defined as limitations in usual activities such as work or other daily activities because of emotional problems; and five items address the domain of general mental health. The single unscaled item asks respondents about their general health compared with 1 year ago.

### SF-36 scoring rules and statistical analysis

Each of the 36 items was scored on a scale from 0 to 100, with 100 representing the most favorable state of health. We then summarized the scores for each item and averaged the scores for items within each domain to produce domain scores. All scoring was performed by a psychiatrist using the scoring algorithm developed by Ware et al (12).

We used a two-tailed *t* test to compare domain scores of the obese and normal-weight groups and considered differences to be significant at α = .05. In addition, we defined a *decrease* in quality of life for an individual as an average score of less than 50 for the eight SF-36 domains. We calculated the percentage of individuals within each group who scored less than 50.

## Results

All individuals surveyed completed the questionnaire in full. Table 1 shows the mean age and BMI by sex for each participant group.

Table 2 shows the mean scores by sex for obese participants and normal-weight participants in each of the eight SF-36 domains. Differences in scores between obese women and normal-weight women were statistically significant in seven of eight domains; the sole exception was for vitality. Differences in scores between obese men and normal-weight men were statistically significant in six of eight domains; the two exceptions were for role-emotional and vitality.

We found that obese women were significantly more impaired than obese men in four of eight domains: role–emotional (*P* = .05), vitality (*P *= .03), bodily pain (*P* = .04), and general health perception (*P* = .001).

Among obese women, 45.0% (674/1498) experienced a reduced quality of life, compared with only 13.2% (33/250) of normal-weight women. Similarly, 41.3% (105/254) of obese men experienced a reduced quality of life, compared with 9.3% (14/150) of normal-weight men.

Table 3 shows how each participant group responded to each SF-36 question.

## Discussion

Obesity is a major public health problem associated with increased health risks (1,3,15-18). The results of our study showed that obesity was related to role-emotional limitations and physical problems. People with obesity uniformly perceive their general health as poorer than healthy-weight people perceive their health (3,18,19). Moreover, a continuum has been observed between mildly, moderately, and severely (morbidly) obese individuals and worsening perceived health status. Some studies found obesity to be associated with compromised quality of life and mental well-being (4,9,18,20,21). Obese people have also been shown to have poorer psychological profiles than other chronically ill people (16), and BMI levels have been found to be positively correlated with reports of self-harm and psychiatric illness (17,22). Investigators also found that higher waist-to-hip ratios are associated with lower socioeconomic status, work problems, unemployment, and increased sedentary behavior among men and women (4,23,24).

Fontaine et al administered the SF-36 questionnaire to 334 people seeking outpatient weight-loss treatment and found that they scored significantly worse than population norms in eight domains; they also found that a morbidly obese subgroup scored significantly worse in six of the eight scales (17). On the other hand, some studies found little difference between obese and nonobese groups in their scores on standard psychological tests (23-25).

Obesity is no doubt, however, associated with some loss of quality of life, particularly in physical well-being (22,26-29). In a population sample of 3443 men and women from the Netherlands, Seidell et al found that BMI was positively associated with the number of health complaints (20). Similarly, Richards et al compared the functional status of 345 sibling pairs, one classified as severely obese (BMI ≥35.0) and the other classified as normal weight (24). All SF-36 functional status and emotional well-being scores were significantly lower among the severely obese participants than among their normal-weight siblings. Furthermore, those who were severely obese perceived their general health to be poorer and more likely to get worse than did their normal-weight siblings (24).

Body-image dissatisfaction (19) and binge-eating disorder (18,30) are both more common among obese people than among normal-weight people (22). Although few obese patients have clinically significant problems with body image, binge-eating disorder is associated with high rates of psychopathology, particularly depression (25). Some obese people suffer from clinically significant psychopathology that requires treatment. Research is needed to examine factors that may increase the risk for psychopathology among the heterogeneous obese population. A recent study suggested that gender may modify the psychological risk of obesity. In a general population sample, excess weight among women was associated with an increased risk for major depression, suicidal thoughts, and suicide attempts (11,28), whereas excess weight among men was associated with a decreased risk for depression and suicidal behavior (28). Similarly, Sullivan et al found that the psychosocial consequences of obesity were greater among women with a BMI greater than 34.0 than among men with a BMI greater than 38.0 (29).

Mathias et al reported that obese patients scored worse than normal-weight individuals on ratings of overweight distress, physical appearance, and health-state preferences (31). Doll et al found a strong inverse relationship between BMI and quality of life (32). In our study, we found obesity to be associated with both poorer quality of life and decreased mental well-being, compared with normal weight. For example, we found that 25.9% of obese women and 16.2% of obese men reported difficulty bathing or dressing themselves; only 7.8% of normal-weight women and no normal-weight men found difficulty with this task. This finding shows that obesity affects physical well-being and the ability to perform daily activities.

Evidence of a negative correlation between obesity and psychological quality of life is equivocal, and it is much weaker than evidence of a negative correlation between obesity and physical quality of life. Earlier studies found few or no differences between obese and normal-weight people in psychological functioning (1,2). Similarly, recent population-based studies demonstrated marked differences between obese and nonobese people in their physical quality of life but few differences between them in their psychological or social quality of life. Nevertheless, there is some good evidence that obesity does negatively affect psychological quality of life (4,32,33).

Studies of self-reported health status show that women are more likely than men to report impaired health (28,30,31,33,34). Similarly, women are more likely than men to report impaired body image or body satisfaction, and obese women are more likely than obese men to report impaired health-related quality of life (35). In our study, we found that obese women were significantly more impaired than obese men in four of the eight quality-of-life domains. The results of our study add to the substantial body of evidence that obesity is associated with poor levels of health status, particularly with poor levels of physical and social well-being.

## Figures and Tables

**Table 1:** Mean (SD) Age and Body Mass Index (BMI) of Participants in Study of Quality of Life Among Obese and Normal-Weight Men and Women, Turkey, 2005

Category	No. Participants	Age, y	BMI
**Obese (BMI ≥30.0)**			
Men	254	40.3 (3.9)	33.7 (3.4)
Women	1498	43.3 (4.4)	36.3 (9.4)
**Normal weight (BMI <25.0)**			
Men	150	44.6 (6.0)	23.3 (4.4)
Women	250	41.1 (8.0)	22.1 (3.8)

**Table 2:** Mean Scores (SD) of Obese and Normal-Weight Men and Women on the Eight Quality-of-Life Domains Measured by the SF-36 Survey, Turkey, 2005^a^

Domain	Women			Men		

	Obese (n = 1498)	Normal Weight (n = 250)	*P* b	Obese (n = 254)	Normal Weight (n = 150)	*P* b
Physical functioning	63.7 (10.5)	85.4 (13.3)	.03	69.9 (8.5)	90.3 (13.7)	.004
Role–physical	44.4 (5.4)	90.5 (13.8)	.003	45.3 (8.4)	89.4 (14.6)	<.001
Social functioning	64.3 (7.3)	85.8 (14.4)	.03	64.4 (13.7)	86.8 (8.3)	.003
Role–emotional	43.9 (4.6)	87.3 (10.9)	.007	76.8 (14.5)	88.3 (10.9)	.73
Mental health	54.7 (8.4)	70.5 (14.6)	.04	59.6 (9.3)	74.4 (13.4)	.04
Vitality	53.8 (14.4)	60.3 (5.8)	.54	65.3 (10.3)	70.3 (10.7)	.09
Bodily pain	50.3 (14.3)	84.3 (13.4)	.03	69.3 (11.4)	80.3 (9.4)	.004
General health perception	43.0 (9.7)	75.9 (16.4)	.004	64.3 (13.3)	79.3 (8.3)	.004

a Obese is defined as a body mass index (BMI) ≥30.0; normal weight, as a BMI <25.0.

b *P *values were determined by two-tailed *t* test.

**Table 3:** SF-36 Survey Results (%) Among Participants in Study of Quality of Life Among Obese and Normal-Weight Men and Women, Turkey, 2005

Survey Item	Obese		Normal Weight	

	Women (n = 1498)	Men (n = 254)	Women (n = 250)	Men (n = 150)
**In general, health is**				
Excellent	9.0	10.3	25.4	29.8
Very good	11.1	18.6	30.1	34.1
Good	25.7	15.1	28.7	20.1
Fair	20.7	29.9	10.1	13.6
Poor	33.5	26.1	5.7	2.4
**General health compared with 1 year ago**				
Much better	2.4	4.9	10.7	15.3
Somewhat better	9.2	8.7	18.6	17.3
About the same	30.7	26.1	37.8	39.1
Somewhat worse	34.9	39.8	20.9	15.9
Much worse	22.8	20.6	12.0	12.4
**Vigorous activities, such as running, lifting heavy objects, participating in strenuous sports**				
Yes, limited a lot	54.9	49.1	43.9	45.7
Yes, limited a little	34.3	42.7	47.1	43.0
No, not limited at all	10.8	8.2	9.0	11.3
**Moderate activities, such as moving a table, pushing a vacuum cleaner, bowling, or playing golf**				
Yes, limited a lot	44.9	41.3	35.9	30.3
Yes, limited a little	46.3	40.5	47.1	51.0
No, not limited at all	8.7	18.2	17.0	18.7
**Climbing several flights of stairs**				
Yes, limited a lot	27.7	13.1	2.9	4.0
Yes, limited a little	38.4	18.1	7.1	3.0
No, not limited at all	33.9	68.8	90.0	93.0
**Climbing one flight of stairs**				
Yes, limited a lot	20.6	19.8	19.1	15.3
Yes, limited a little	35.1	34.1	32.3	27.6
No, not limited at all	44.3	46.1	47.6	57.1
**Walking several blocks**				
Yes, limited a lot	35.1	33.9	29.7	21.0
Yes, limited a little	38.4	48.4	51.9	51.1
No, not limited at all	26.6	17.7	18.4	27.8
**Walking one block**				
Yes, limited a lot	28.6	30.6	30.7	31.0
Yes, limited a little	45.1	51.7	43.5	51.1
No, not limited at all	26.3	17.7	26.8	17.8
**Walking more than a mile**				
Yes, limited a lot	40.7	33.1	28.3	27.5
Yes, limited a little	25.1	45.9	40.0	46.9
No, not limited at all	34.2	22.0	31.7	26.6
**Lifting or carrying groceries**				
Yes, limited a lot	21.1	9.9	4.4	2.0
Yes, limited a little	33.9	13.8	10.8	8.0
No, not limited at all	45.0	76.3	84.8	90.0
**Bending, kneeling, or stooping**				
Yes, limited a lot	20.2	20.0	3.0	2.0
Yes, limited a little	30.0	20.0	2.6	1.1
No, not limited at all	49.8	60.0	94.4	96.9
**Bathing or dressing**				
Yes, limited a lot	10.6	4.1	3.7	0
Yes, limited a little	15.3	12.1	4.1	0
No, not limited at all	74.1	83.8	92.2	100.0
**Difficulty performing work or other activities**				
Yes	65.9	60.9	59.8	60.6
No	34.1	39.1	40.2	38.4
**Limited in the kind of work or other activities**				
Yes	65.6	50.1	54.9	47.1
No	34.4	49.9	46.1	52.9
**Accomplished less than would like at work or other activities (as a result of physical problems)**				
Yes	39.6	40.3	20.3	17.5
No	60.4	59.7	79.7	82.5
**Cut down on time spent on work or other activities (as a result of physical problems)**				
Yes	46.6	30.3	21.6	17.9
No	53.4	69.7	78.4	82.1
**During the past 4 weeks, accomplished less than would like at work or other activities (as a result of emotional problems)**				
Yes	49.6	40.3	23.8	19.8
No	50.4	59.7	76.2	80.2
**Didn't do work or other activities as carefully as usual (as a result of emotional problems)**				
Yes	31.4	20.2	11.3	6.1
No	68.6	79.8	88.7	93.9
**Cut down on time spent on work or other activities (as a result of emotional problems)**				
Yes	46.6	30.1	29.6	18.5
No	53.4	69.9	70.4	81.5
**During the past 4 weeks, physical or emotional problems have interfered with social activities**				
Not at all	15.4	19.4	40.1	43.7
Slightly	18.6	30.6	28.6	30.2
Moderately	53.3	31.1	23.3	13.3
Quite a bit	10.6	9.7	7.5	12.0
Extremely	2.1	9.2	0.5	0.8
**How much bodily pain during the past 4 weeks?**				
None	16.1	20.8	32.0	25.1
Very mild	28.1	25.6	33.6	41.9
Mild	26.9	37.1	10.8	17.1
Moderate	24.3	14.3	19.6	15.5
Severe	4.6	2.1	4.0	0.3
**Pain interfered with normal work**				
Not at all	10.1	24.6	40.2	50.1
A little bit	15.0	43.8	30.0	25.3
Moderately	66.3	20.3	20.3	10.9
Quite a bit	8.0	11.0	9.0	13.0
Extremely	0.7	0.3	0.5	0.7
**Felt full of pep**				
All of the time	6.1	7.6	10.1	8.9
Most of the time	15.0	13.5	20.9	10.8
A good bit of the time	24.3	25.3	31.3	20.8
Some of the time	9.3	13.8	26.2	30.7
A little of the time	45.0	30.0	10.6	15.9
None of the time	0.3	9.8	0.9	12.9
**Have been a happy person**				
All of the time	7.6	14.6	30.1	24.1
Most of the time	15.0	23.8	20.0	28.6
A good bit of the time	16.1	25.3	21.3	15.8
Some of the time	50.3	33.8	27.0	27.0
A little of the time	8.9	2.0	1.6	4.0
None of the time	2.1	0.5	0	0.5
**Have felt tired**				
All of the time	5.1	4.9	4.9	3.1
Most of the time	4.9	6.9	5.0	7.1
A good bit of the time	20.5	30.1	22.9	12.0
Some of the time	44.3	29.7	14.5	15.6
A little of the time	25.1	28.4	17.1	21.9
None of the time	0	0	35.6	40.3
**Have been a very nervous person**				
All of the time	4.1	7.8	2.9	3.9
Most of the time	5.5	9.7	6.6	9.0
A good bit of the time	10.4	20.1	11.3	22.0
Some of the time	34.3	39.3	14.5	12.9
A little of the time	25.0	13.1	37.1	32.5
None of the time	20.7	10.0	27.6	19.7
**Have felt calm and peaceful**				
All of the time	24.5	27.9	32.9	30.1
Most of the time	25.5	19.1	26.6	29.0
A good bit of the time	10.3	17.9	11.8	12.5
Some of the time	20.8	19.3	8.1	9.7
A little of the time	9.0	12.1	15.3	5.8
None of the time	9.9	3.7	5.3	12.9
**Have felt downhearted and blue**				
All of the time	14.1	19.5	12.3	19.5
Most of the time	19.4	19.1	17.7	15.8
A good bit of the time	20.9	23.4	19.6	31.2
Some of the time	22.0	15.9	11.1	18.4
A little of the time	15.7	12.1	18.8	8.2
None of the time	7.9	10.0	20.5	6.9
**Have felt down in the dumps**				
All of the time	10.5	9.8	10.9	9.4
Most of the time	15.5	19.1	13.6	8.0
A good bit of the time	10.1	15.6	14.8	22.8
Some of the time	24.3	29.3	15.9	17.9
A little of the time	25.0	13.0	16.9	12.5
None of the time	14.6	13.2	27.9	29.4
**Have a lot of energy**				
All of the time	5.5	3.8	18.9	15.1
Most of the time	8.5	17.6	13.6	23.9
A good bit of the time	20.1	17.1	17.8	27.1
Some of the time	34.3	29.3	30.9	21.5
A little of the time	28.3	23.0	14.8	10.0
None of the time	3.3	9.2	4.0	2.4
**Have felt worn out**				
All of the time	7.1	8.9	9.9	10.1
Most of the time	14.9	10.6	9.6	12.9
A good bit of the time	21.8	19.4	11.8	29.1
Some of the time	25.9	32.8	40.9	16.0
A little of the time	19.1	20.6	24.8	30.8
None of the time	11.2	7.7	3.0	1.1
**During the past 4 weeks, how much of the time has physical health or emotional problems interfered with social activities?**				
All of the time	13.6	11.5	7.1	3.6
Most of the time	18.1	17.4	14.1	5.9
A good bit of the time	18.5	20.3	18.1	19.7
Some of the time	23.1	25.2	21.9	22.9
A little of the time	17.2	19.9	24.8	31.8
None of the time	9.5	5.7	14.0	16.1
**Health to get worse**				
Definitely true	7.5	6.9	5.1	2.1
Mostly true	32.1	30.1	12.6	6.0
Don't know	22.8	29.9	31.3	42.5
Mostly false	28.7	25.3	38.1	39.4
Definitely false	8.9	7.8	12.9	10.0
**Get sick a little easier than other people**				
Definitely true	15.3	1.5	7.0	8.0
Mostly true	28.9	23.1	10.7	11.0
Don't know	35.8	39.9	21.3	15.5
Mostly false	12.9	26.4	38.6	39.4
Definitely false	7.1	9.1	22.4	26.1
**As healthy as anybody I know**				
Definitely true	31.2	40.1	21.7	18.4
Mostly true	20.6	20.5	15.4	12.0
Don't know	21.8	18.4	25.1	23.1
Mostly false	12.3	11.3	18.3	22.5
Definitely false	14.1	9.7	19.5	24.0
**Health is excellent**				
Definitely true	10.4	10.6	21.8	28.9
Mostly true	33.4	21.8	25.3	23.8
Don't know	30.1	28.7	21.3	23.9
Mostly false	11.4	27.4	15.4	11.3
Definitely false	14.7	11.5	16.2	12.1

Some categories do not add to 100% because of rounding.

a Obese is defined as a body mass index (BMI) ≥30.0; normal weight, as a BMI <25.0.
